# Two different network topologies yield bistability in models of mesoderm and anterior mesendoderm specification in amphibians

**DOI:** 10.1016/j.jtbi.2014.03.015

**Published:** 2014-07-21

**Authors:** L.E. Brown, J.R. King, M. Loose

**Affiliations:** a MyCIB, School of Biosciences, University of Nottingham, Sutton Bonington LE12 5RD, UK; b School of Mathematical Sciences, University of Nottingham, University Park, Nottingham NG7 2RD, UK; c Centre for Genetics and Genomics, University of Nottingham, Queen׳s Medical Centre, Nottingham NG7 2UH, UK

**Keywords:** Embryo development, Network motifs, Cell differentiation, Gene regulatory networks

## Abstract

Understanding the Gene Regulatory Networks (GRNs) that underlie development is a major question for systems biology. The establishment of the germ layers is amongst the earliest events of development and has been characterised in numerous model systems. The establishment of the mesoderm is best characterised in the frog *Xenopus laevis* and has been well studied both experimentally and mathematically. However, the *Xenopus* network has significant differences from that in mouse and humans, including the presence of multiple copies of two key genes in the network, Mix and Nodal. The axolotl, a urodele amphibian, provides a model with all the benefits of amphibian embryology but crucially only a single Mix and Nodal gene required for the specification of the mesoderm. Remarkably, the number of genes within the network is not the only difference. The interaction between Mix and Brachyury, two transcription factors involved in the establishment of the endoderm and mesoderm respectively, is not conserved. While Mix represses Brachyury in *Xenopus*, it activates Brachyury in axolotl. Thus, whilst the topology of the networks in the two species differs, both are able to form mesoderm and endoderm *in vivo*. Based on current knowledge of the structure of the mesendoderm GRN we develop deterministic models that describe the time evolution of transcription factors in a single axolotl cell and compare numerical simulations with previous results from *Xenopus*. The models are shown to have stable steady states corresponding to mesoderm and anterior mesendoderm, with the *in vitro* model showing how the concentration of Activin can determine cell fate, while the *in vivo* model shows that *β*-catenin concentration can determine cell fate. Moreover, our analysis suggests that additional components must be important in the axolotl network in the specification of the full range of tissues.

## Highlights

• We present models of mesendoderm specification in the urodele amphibian, the axolotl.
• *in vitro* and *in vivo* models are simulated and compared with experimental data.
• The model topology differs from that of the anuran amphibian, *Xenopus laevis*.
• Steady states representing mesoderm and anterior mesendoderm are found in both models.
• Both the axolotl and *Xenopus* topologies can account for similar qualitative data.

## 1 Introduction

Whilst far from the sole determinant of how cell types are specified, the interplay of transcription factors (TFs) and signalling molecules to form networks that regulate cell fate provides a program that underlies the development of an organism. TFs bind to promoter sites localised within target genes either to up- or to down-regulate their expression. Target genes may themselves produce TFs or signalling molecules which are secreted by the cell to activate signalling cascades and activate intracellular transducers that in turn activate target genes and so form gene regulatory networks (GRNs) driving development. During the development of an embryo from a single cell (the fertilised egg) to a fully developed multicellular adult organism, cells differentiate into increasingly specialised cell fates. The timing and location of gene expression, as regulated by developmental GRNs, ensures that an embryo develops to form the correct body plan in the adult organism. One of the earliest events in embryo development is the formation of the three primary germ layers, mesoderm, endoderm and ectoderm (Gilbert, 2010; Slack, 1991). Each germ layer gives rise to different tissue types in the developing embryo: endoderm (the inner layer) forms the digestive system and the lungs, mesoderm (the middle layer) forms muscle, blood and connective tissue and ectoderm (the outer layer) forms the epidermis and nervous system. Collectively, the cells giving rise to both mesoderm and endoderm have been named as the mesendoderm. The specification of these cells is amongst the earliest events in the embryo and so is easily investigated experimentally.

The GRN governing the specification of mesoderm and endoderm, here termed the mesendoderm GRN (mGRN), has been studied in several species including *Caenorhabditis elegans* (Maduro, 2006), *Strongylocentrotus purpuratus* (sea urchin) (Davidson et al., 2002), *Xenopus laevis* (Loose and Patient, 2004; Koide et al., 2005) and *Ambystoma mexicanum* (Swiers et al., 2010). Notably, both *Xenopus laevis* (the frog) and *Ambystoma mexicanum* (the axolotl) are amphibians although from different orders. The frog (an anuran) and the axolotl (a urodele) differ in several significant aspects of development (Johnson et al., 2011). Remarkably these differences extend to the topology of the mGRN with significant differences in the interactions between key TFs having been identified (Swiers et al., 2010). Several mathematical models based on the *Xenopus* mGRN have been developed and analysed to provide greater understanding of how mesendoderm forms (Saka and Smith, 2007; Middleton et al., 2009). Saka and Smith (2007) show that a simple negative feedback loop can reproduce experimental observations, providing a possible mechanism for the formation of different cell types. Middleton et al. (2009) base their model on a simplified version of the *Xenopus* mGRN, representing large *Mix* and *Nodal* gene families by a single node for each gene, motivated by single copies of these genes in mammals (Guo et al., 2002; Zhou et al., 1999). The model describes the time evolution of each gene in the simplified GRN in a single cell, neglecting spatial effects. The model is able to reproduce qualitatively Activin and VegT dose response experiments, with stable steady states of the model corresponding to mesoderm and anterior mesendoderm cell fates. An important interaction for producing this behaviour is the mutual negative regulation of *Mix* and *Brachyury*. In contrast with *Xenopus*, the genome of the axolotl contains only a single *Mix* gene and two *Nodal* genes, with only one of these required for mesendoderm formation (Swiers et al., 2010). A study of the evolutionary history of *Nodal* genes suggests that ancestral species have two *Nodal* genes while higher vertebrates have lost one (Hellsten et al., 2010). Thus the expanded Mix and Nodal families in *Xenopus* are likely to be a divergent trait. Intriguingly, the relationship between *Mix* and *Brachyury* is not conserved between *Xenopus* and axolotl. The mutual negative feedback between *Mix* and *Brachyury* in *Xenopus* is key to the establishment of the mesoderm and the endoderm and so caused us to question if an alternate topology was still able to establish distinct germ layers during development. This is of particular importance as Swiers et al. (2010) show that the axolotl network, not that of *Xenopus*, is conserved with the mouse. In this paper we formulate mathematical models based on the axolotl mGRN and compare them qualitatively with the *Xenopus* mGRN, showing that the axolotl network topology can specify mesoderm and anterior mesendoderm cell fates.

## 2 Biological background

### 2.1 Mesoderm and endoderm formation in *Xenopus*

The GRN underlying the formation of mesoderm and endoderm in the anuran amphibian *Xenopus laevis* described in Loose and Patient (2004) and Koide et al. (2005) contains around 50 TFs and signals. Important genes within the *Xenopus* mGRN include the maternal factors VegT and *β*-catenin and downstream factors *Mix*.1, *Brachyury*, *Goosecoid* and the *Nodal* family. The maternal factors VegT and *β*-catenin provide initial positional information and initiate the expression of genes, including members of the *Nodal* gene family. Embryos depleted of VegT fail to form endoderm (Zhang et al., 1998; Kofron et al., 1999) and mesoderm (Kofron et al., 1999). The ability of VegT to induce mesoderm and endoderm is via its regulation of TGF-*β* (*Nodal*) signalling (Clements et al., 1999; Kofron et al., 1999). VegT can also directly activate *Mix*.1 and *Brachyury* (Loose and Patient, 2004). *β*-catenin is expressed in the dorsal region of the embryo following an event known as cortical rotation (Weaver and Kimelman, 2004), and by stage 9.5 its expression has spread around an equatorial ring in the prospective mesoderm (Schohl and Fagotto, 2002). Both the knockdown and the overexpression of *β*-catenin reveal that it regulates expression of mesodermal genes such as *Brachyury* (Schohl and Fagotto, 2003) and Nodal signalling, affecting the temporal pattern but not the overall levels of P-Smad2 activation (Lee et al., 2001). The ability of *Nodal* genes to induce mesoderm and endoderm has been investigated using Activin, an agonist of Nodal signalling. Dose response experiments (Gurdon et al., 1996, 1999; Papin and Smith, 2000; Gurdon et al., 1994; Green and Smith, 1990) show that at low concentrations of Activin a cell will become mesoderm (i.e. will express *Brachyury*). As the dose of Activin increases past a threshold value, a cell will no longer express *Brachyury* but will express *Mix*.1 (i.e. endoderm). Note that Activin is not expressed at the correct time or location to act in mesoderm and endoderm induction *in vivo* and that *Nodal-related* genes are the prime candidates for the morphogens regulating the induction of mesoderm and endoderm in *Xenopus*. FGF signals have a role in maintaining Brachyury expression in mesoderm, with a positive autoregulatory feedback loop between Brachyury and FGF (Isaacs et al., 1994). FGF also has a role in ectodermal cell fates, in particular in specifying neural tissue. Low levels of FGF in combination with an inhibition of BMP result in neural cell fates, while high levels of FGF in combination with *Nodal-related* genes result in mesodermal cell fates (Delaune et al., 2005). However, here we consider only the mGRN, which neglects factors involved in ectoderm formation, as such we consider only the positive feedback between FGF and Brachyury and neglect the role of FGF in specifying neural fates.

Cell types can be identified by the genes they express: *Brachyury* expressing cells correspond to mesoderm and *Mix*.1 expressing cells to endoderm (Lemaire, 1998). *Goosecoid* expressing cells correspond to dorsal mesoderm, which gives rise to anterior (head forming) structures (Cho et al., 1991). In this paper, we refer to cells co-expressing *Goosecoid* and *Mix*.1 as anterior mesendoderm, which forms in dorsal regions of the embryo. An important interaction in the full mGRN (Loose and Patient, 2004) is the mutual repression of *Mix*.1 and *Brachyury* (Lemaire, 1998; Latinkic and Smith, 1999) creating competition between mesoderm and endoderm which is thought to contribute to the separation of these two germ layers (Lemaire, 1998).

### 2.2 The axolotl mesendoderm GRN

**Fig. 1:**
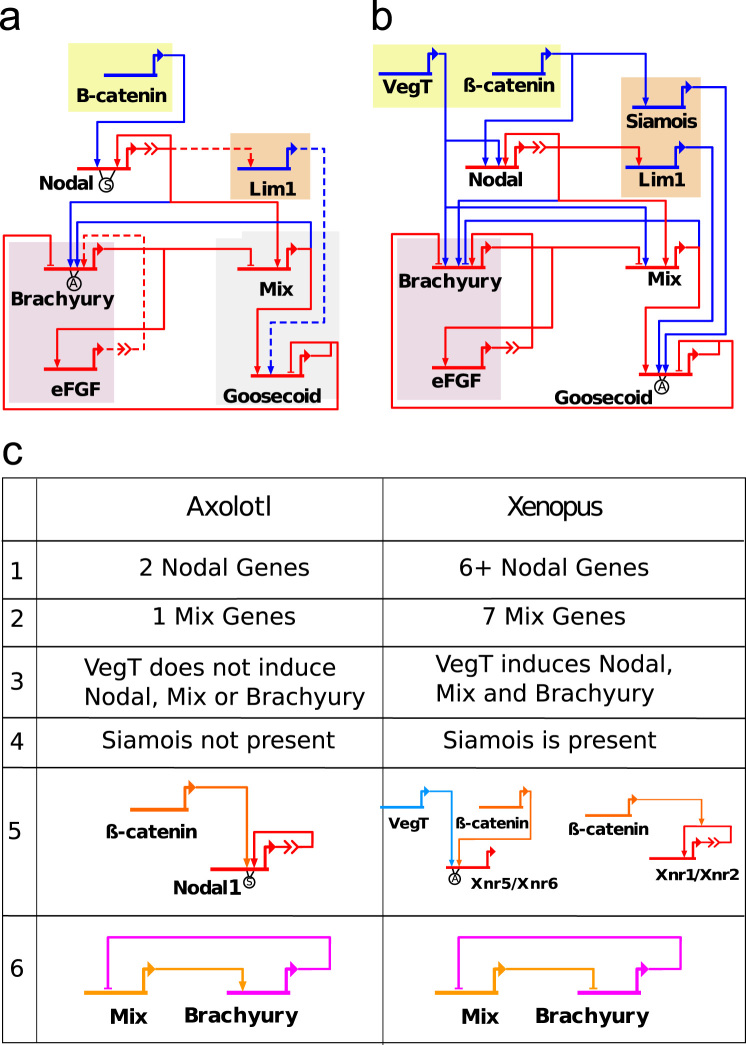
A comparison of the axolotl and *Xenopus* mesendoderm GRNs. (a) The axolotl mesendoderm GRN and (b) the simplified *Xenopus* mesendoderm GRN. Arrow and bar heads represent, respectively, activation and repression. The ‘A’ indicates that an input is, in Boolean terms, an ‘AND’ gate. The ‘S’ indicates a synergy between the two transcription factors, i.e. *β*-catenin activates Nodal1 and this activation is enhanced by Nodal autoregulation. Otherwise, multiple inputs consisting of only one type (repression or activation) correspond to an ‘OR’ gate. When both types are present, the repression and activation inputs are treated as two ‘OR’ gates coupled by an ‘AND’ gate. Red lines show interactions which are the same in both networks and blue lines show those which differ. In (b) solid lines indicate experimentally verified links and dashed lines indicate links which are inferred from the *Xenopus* mesendoderm network, and which need to be verified experimentally. (c) Table summarising the main differences between the axolotl and *Xenopus* mesendoderm GRNs. Row 1: At least 6 Nodal genes are found in *Xenopus*, compared with 2 Nodal genes in axolotl. Row 2: There are seven Mix genes in *Xenopus* and one Mix gene in axolotl. Row 3: VegT acts to activate expression of Nodal, Mix and Brachyury in *Xenopus*, but in axolotl VegT does not activate these genes. Row 4: *Siamois* is a gene found in *Xenopus* but not axolotl. Row 5: In *Xenopus*, *β*-catenin acts in two different ways on Nodal: *β*-catenin enhances Nodal autoregulation of Xnr1 and Xnr2, and the expression of Xnr5 and Xnr6 is activated by *β*-catenin in the presence of VegT. In axolotl, Nodal1 can be activated by *β*-catenin alone and we also assume that it can enhance Nodal autoregulation. Row 6: Mix and Brachyury mutually repress each other in *Xenopus*, but, in axolotl, Mix is required for the expression of Brachyury. (For interpretation of the references to colour in this figure caption, the reader is referred to the web version of this paper.)

In addition to the number of members of the *Mix* and *Nodal* gene families, there are several other differences in the topology of the axolotl and *Xenopus* mGRNs illustrated in Fig. 1(c) and discussed here. In *Xenopus*, VegT is localised to the vegetal pole of the oocyte while it is expressed uniformly throughout the oocyte in axolotl (Nath and Ellison, 2007). VegT is also found throughout the embryo in lungfish and sturgeon, suggesting that the localisation of VegT is not an ancestral vertebrate trait (Chen, 2010). Furthermore, whilst VegT can induce *Nodal*, *Mix* and *Brachyury* in *Xenopus* (Loose and Patient, 2004), VegT does not act on these genes in axolotl (Chen, 2010). The induction of *Nodal* by *β*-catenin varies between axolotl and *Xenopus* and also between different Nodal genes in *Xenopus*. Axolotl *Nodal*1 is activated as a direct downstream target of *β*-catenin (Chen, 2010). It is also thought that *β*-catenin may act to enhance Nodal autoregulation, as is seen for the *Xenopus Nodal* genes *Xnr*1 and *Xnr*2 (Loose and Patient, 2004), but this has yet to be tested experimentally in axolotls. The *Xenopus* Nodal genes *Xnr*5 and *Xnr*6 require both VegT and *β*-catenin to be present to be transcribed (Takahashi et al., 2000; Loose and Patient, 2004). *Siamois*, a gene expressed in the organiser region of the embryo, appears to be specific to *Xenopus* with no similar gene found in fish or amniotes (Hellsten et al., 2010). Our own extensive searches for a *Siamois* ortholog in the axolotl suggest that this gene is an anuran innovation (data not shown). Perhaps most significantly, Mix is required in the axolotl for the expression of *Brachyury* while Brachyury represses *Mix*. This contrasts with *Xenopus* where *Mix*.1 and *Brachyury* negatively regulate one another׳s expression. Our current knowledge of the axolotl mGRN is described in Fig. 1(a). Solid lines in this network are experimentally verified whilst dashed lines are interactions which have been inferred from the *Xenopus* mGRN and are in accord with our current understanding of the axolotl network. Notwithstanding these topological differences, both axolotl and *Xenopus* embryos form all three germ layers, demonstrating that both networks are capable of supporting differentiation.

**Fig. 2:**
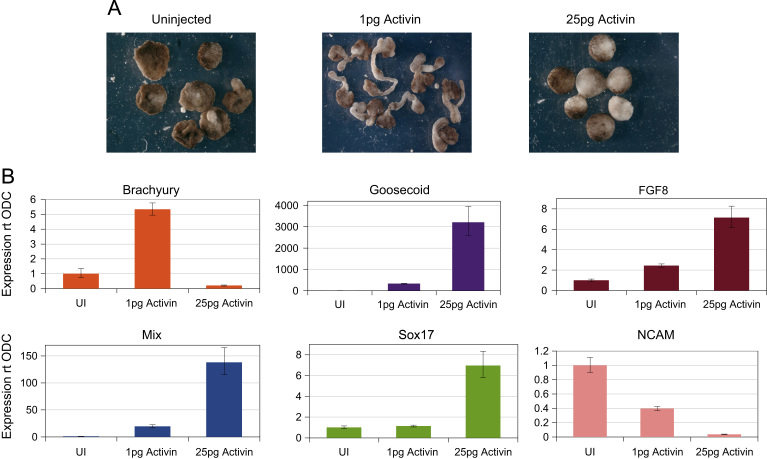
Mesoderm and anterior mesendoderm induction by Activin in animal cap explants (48 h after animal caps explants are cut from embryo). (A) Axolotl animal caps injected with 1pg Activin mRNA induce mesoderm, and 25pg of Activin induces anterior mesendoderm. (B) qPCR analysis of Brachyury, Mix, Sox17, Goosecoid, FGF8 and NCAM expression in animal caps.

Since Activin has been shown to induce mesoderm and endoderm in a dose dependent manner in *Xenopus* animal caps, we tested its ability to do the same in axolotl animal caps (Fig. 2). As previously shown (Swiers et al., 2010), 1 pg of RNA encoding Activin causes axolotl animal caps to elongate, indicating that mesoderm has been induced, as confirmed by the induction of Brachyury. Animal caps injected with 25 pg Activin RNA show a phenotype associated with endoderm. An analysis of gene expression in these caps shows that they express the endoderm specific marker Sox17 and also Mix and Goosecoid. An analysis of the expression of a neural specific marker NCAM show that it is expressed at lower levels in Activin-injected caps when compared with uninjected levels, suggesting no unexpected neural induction has occurred. Thus, as previously shown in *Xenopus*, Activin can also induce mesoderm and endoderm in a dose dependent manner in axolotl.

We now propose mathematical models for the axolotl mGRN, based on current experimental knowledge of mesoderm and endoderm specification in axolotl. We develop both an *in vitro* model in which Activin is used as a stimulus of Nodal signalling and an *in vivo* model where the maternal factor *β*-catenin activates Nodal signalling which in turn regulates the expression of downstream targets. A qualitative analysis of the models is carried out to investigate whether, given the changes between the axolotl and *Xenopus* mGRNs, the axolotl models can form mesoderm and anterior mesendoderm in accord with experimental data.

## 3 Model formulation

In this section we formulate sets of governing equations for the axolotl mGRN. Our approach is similar to that of Middleton et al. (2009), where the *in vitro* model describes the mGRN in a single dissociated cell downstream of Activin and the *in vivo* model describes the mGRN downstream of the maternal factor *β*-catenin in a single cell embedded in a population of uniform cells. The *in vitro* model assumes that signals (eFGF and Nodal) secreted by a single cell are too weak to act on downstream targets, but are included in the *in vivo* model. A first order ODE is formulated describing the time evolution of each species in the mGRN, based on the underlying logic of the network.

### 3.1 The axolotl *in vitro* model

**Fig. 3:**
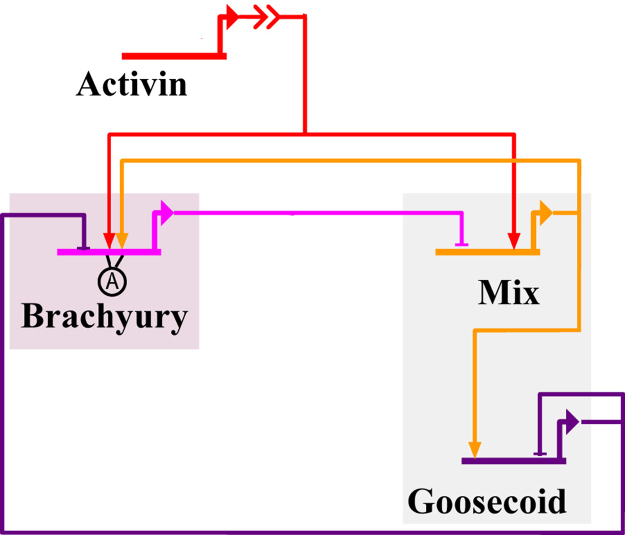
The axolotl *in vitro* network: Nodal signalling is simulated by bathing either whole or dissociated animal caps in Activin. Note that this network is identical to the simplified *Xenopus in vitro* network, except that Mix is required here for the expression of Brachyury.

The axolotl *in vitro* model governs the time evolution of Brachyury (*B*), Mix (*M*) and Goosecoid (*G*) concentrations downstream of Activin (*A*) in a single dissociated cell, based on the mGRN shown in Fig. 3. In the *Xenopus in vitro* model (Middleton et al., 2009) it was assumed that the input of Activin is a constant parameter, supported by biological evidence in *Xenopus* that a cell can remember the concentration of Activin it is initially exposed to via the maintenance of a pool of phosphorylated Smad (Bourillot et al., 2002). We assume that this memory of the level of Activin is a general feature of Activin signalling in all species and thus set the Activin level (*A*) to be constant here. The notations used are as follows: *X* is the concentration of the protein product $\overset{¯}{X}$ of gene X. The rate of production of X induced by $\overset{¯}{Y}$ and the rate of turnover of X are given by positive constants $\lambda_{Y\cdot X}$ and $\mu_{X}$, respectively. A summary of notations is given in Table 1. The governing equations are taken to be

(1a) $$\frac{\mathrm{dB}}{\mathrm{dt}}=\lambda_{A,B}H(\frac{A}{\theta_{A,B}})H(\frac{M}{\theta_{M,B}})\{1-H(\frac{G}{\theta_{G,B}})\}-\mu_{B}B,$$

(1b) $$\frac{\mathrm{dG}}{\mathrm{dt}}=\lambda_{M,G}H(\frac{M}{\theta_{M,G}})\{1-H(\frac{G}{\theta_{G,G}})\}-\mu_{G}G,$$

(1c) $$\frac{\mathrm{dM}}{\mathrm{dt}}=\lambda_{A,M}H(\frac{A}{\theta_{A,M}})\{1-H(\frac{B}{\theta_{B,M}})\}-\mu_{M}M,$$

where H is the Hill function.

$$H(x)=\frac{x^{m}}{x^{m}+1},$$

with the Hill coefficient m≥1 being a measure of cooperativity of TF-DNA binding (see Alon, 2007; Middleton, 2007 for more details). Initial conditions are selected to represent the state of an animal cap cell which has not yet been treated with Activin, such that Brachyury, Mix and Goosecoid are absent:

(2) B(0)=M(0)=G(0)=0.

Mix, Brachyury and Goosecoid can then only be expressed once the cell is treated with Activin, i.e. A>0.

### 3.2 The axolotl *in vivo* model

The axolotl *in vivo* model includes the time evolution of the maternal factor *β*-catenin (*C*) and the zygotic factors Nodal (*N*), Mix (*M*), Brachyury (*B*), eFGF (*E*), Goosecoid (*G*) and Lim-1 (*L*), based on the network shown in Fig. 1(a). *β*-catenin is treated as an intracellular deposit of protein which turns over at a constant rate. We assume that the Nodal and eFGF signals act in the same way as a transcription factor (i.e. acting directly on their downstream targets). The resulting governing equations are given by

(3a) $$\frac{\mathrm{dC}}{\mathrm{dt}}=-\mu_{C}C,$$

(3b) $$\frac{\mathrm{dN}}{\mathrm{dt}}=\lambda_{C,N}H(\frac{C}{\theta_{C,N}})+\lambda_{N,N}H(\frac{N}{\theta_{N,N}})\{1+\lambda_{C,N2}H(\frac{C}{\theta_{C2,N}})\}-\mu_{N}N,$$

(3c) $$\frac{\mathrm{dL}}{\mathrm{dt}}=\lambda_{N,L}H(\frac{N}{\theta_{N,L}})-\mu_{L}L,$$

(3d) $$\frac{\mathrm{dE}}{\mathrm{dt}}=\lambda_{B,E}H(\frac{B}{\theta_{B,E}})-\mu_{E}E,$$

(3e) $$\frac{\mathrm{dB}}{\mathrm{dt}}=\{\lambda_{E,B}H(\frac{E}{\theta_{E,B}})+\lambda_{\mathrm{NM},B}H(\frac{N}{\theta_{X,B}})H(\frac{M}{\theta_{M,B}})\}\{1-H(\frac{G}{\theta_{G,B}})\}-\mu_{B}B,$$

(3f) $$\frac{\mathrm{dG}}{\mathrm{dt}}=\{\lambda_{L,G}H(\frac{L}{\theta_{L,G}})+\lambda_{M,G}H(\frac{M}{\theta_{M,G}})\}\{1-H(\frac{G}{\theta_{G,G}})\}-\mu_{G}G,$$

(3g) $$\frac{\mathrm{dM}}{\mathrm{dt}}=\lambda_{N,M}H(\frac{N}{\theta_{N,M}})\{1-H(\frac{B}{\theta_{B,M}})\}-\mu_{M}M.$$

An initial concentration of *β*-catenin is required in order for mesoderm and anterior mesendoderm to form such that $C(0)=C_{0}$, where *C*_0_ is a positive constant. All other TFs and signals are initially absent from the cell:

(4) $$C(0)=C_{0},\;N(0)=0,\;L(0)=0,\;E(0)=0,\;B(0)=0,\;G(0)=0,\;M(0)=0.$$

### 3.3 Nondimensionalisation

We nondimensionalise the mathematical models using the time scale of Brachyury turnover, $\tau =\mu_{B}t$. The following threshold parameters are defined for notational simplicity:

(5) $$\theta_{X}\equiv \theta_{X,B},\;\theta_{G}\equiv \theta_{G,B},\;\theta_{B}\equiv \theta_{B,E},\;\theta_{E}\equiv \theta_{E,B},\;\theta_{L}\equiv \theta_{L,G},\;\theta_{M}\equiv \theta_{M,B},\;\theta_{C}\equiv \theta_{C,N},$$

where *X*=*A* in the *in vitro* model and *X*=*N* in the *in vivo* model. Concentrations, *Z*, are scaled Z^=Z/θZ, the initial concentration of *β*-catenin, *C*_0_, is scaled C^0=C0/θC and the dimensionless parameters are

(6) θ^Z,X≡θZ,X/θZ,λ^Y,Z≡λY,Z/θZμB,μ^Z≡μZ/μB.

After applying the non-dimensional scalings (and dropping the hats for notational simplicity) the nondimensional equations governing the systems are given as below. Experimental values of model parameters (thresholds (*θ*), rates of production (*λ*) and decay rates (*μ*)) are not currently available in the literature for either *Xenopus* or axolotl. Without experimental measurements for these parameters, we seek to investigate the qualitative dynamics of the system and explore how changing parameter values affects the behaviour of the system. Parameters are selected such that (7)–(9) are bistable with stable steady states which could be interpreted as mesoderm and anterior mesendoderm. Unless otherwise stated time dependent solutions are computed using the ode15s routine in Matlab and steady state solutions are computed using Xppaut (Ermentrout, 2002).

#### 3.3.1 The axolotl *in vitro* model

The axolotl *in vitro* model is governed by the nondimensional equations:

(7a) $$\frac{\mathrm{dB}}{d\tau }=\lambda_{\mathrm{AM},B}H(A)H(M)\{1-H(G)\}-B,$$

(7b) $$\frac{\mathrm{dG}}{d\tau }=\lambda_{M,G}H(\frac{M}{\theta_{M,G}})\{1-H(\frac{G}{\theta_{G,G}})\}-\mu_{G}G,$$

(7c) $$\frac{\mathrm{dM}}{d\tau }=\lambda_{A,M}H(\frac{A}{\theta_{A,M}})\{1-H(\frac{B}{\theta_{B,M}})\}-\mu_{M}M,$$

subject to initial conditions

(8) M(0)=B(0)=G(0)=0.

#### 3.3.2 The axolotl *in vivo* model

The nondimensional equations governing the axolotl *in vivo* model are given by

(9a) $$\frac{\mathrm{dC}}{d\tau }=-\mu_{C}C,$$

(9b) $$\frac{\mathrm{dN}}{d\tau }=\lambda_{C,N}H(C)+\lambda_{N,N}H(\frac{N}{\theta_{N,N}})\{1+\lambda_{C,N}H(\frac{C}{\theta_{C,N2}})\}-\mu_{N}N,$$

(9c) $$\frac{\mathrm{dL}}{d\tau }=\lambda_{N,L}H(\frac{N}{\theta_{N,L}})-\mu_{L}L,$$

(9d) $$\frac{\mathrm{dE}}{d\tau }=\lambda_{B,E}H(B)-\mu_{E}E,$$

(9e) $$\frac{\mathrm{dB}}{d\tau }=\{\lambda_{E,B}H(E)+\lambda_{\mathrm{NM},B}H(N)H(M)\}\{1-H(G)\}-B,$$

(9f) $$\frac{\mathrm{dG}}{d\tau }=\{\lambda_{L,G}H(L)+\lambda_{M,G}H(\frac{M}{\theta_{M,G}})\}\{1-H(\frac{G}{\theta_{G,G}})\}-\mu_{G}G,$$

(9g) $$\frac{\mathrm{dM}}{d\tau }=\lambda_{N,M}H(\frac{N}{\theta_{N,M}})\{1-H(\frac{B}{\theta_{B,M}})\}-\mu_{M}M,$$

subject to initial conditions

(10) $$C(0)=C_{0},\;N(0)=0,\;L(0)=0,\;E(0)=0,\;B(0)=0,\;G(0)=0,\;M(0)=0.$$

## 4 The axolotl *in vitro* model

### 4.1 Steady-state analysis

**Fig. 4:**
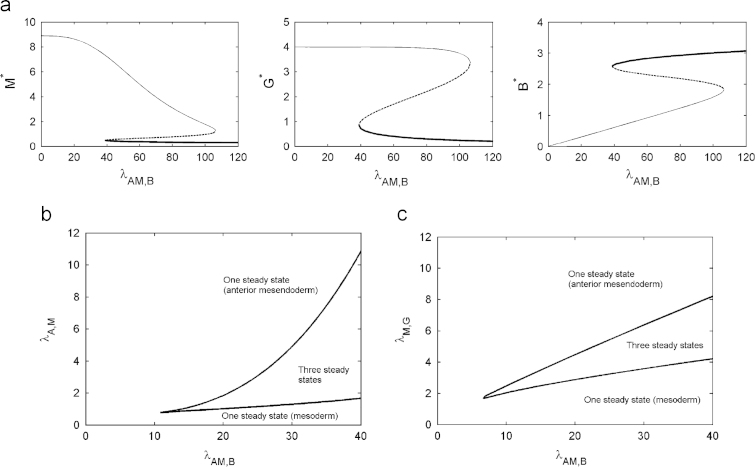
(a) Steady state solutions to (7) plotted against $\lambda_{\mathrm{AM},B}$ for *A*=5. Thick solid lines represent the mesoderm steady state, thin solid lines represent the anterior mesendoderm steady state and dashed lines represent the unstable steady state. Fold bifurcations mark the appearance and the disappearance of the steady states. (b) Solution structure in terms of the bifurcation parameters $\lambda_{\mathrm{AM},B}$ and $\lambda_{A,M}$, these representing the folds that determine the maximum rates of production of Brachyury and Mix in response to activation by Activin. (c) Solution structure in terms of the bifurcation parameters $\lambda_{\mathrm{AM},B}$ and $\lambda_{M,G}$, these representing the folds that determine the maximum rates of production of Brachyury in response to activation by Activin and Goosecoid in response to Mix. Unless otherwise stated, parameters were chosen as in Table 2.

In this section we are concerned with the steady states of the axolotl *in vitro* model (i.e. the system (7)). In particular, we investigate if, given the change in the regulation of Brachyury compared with the *Xenopus in vitro* model of Middleton et al. (2009), stable steady states corresponding to mesoderm and anterior mesendoderm are solutions to the system. We manually tuned parameters of the form $\lambda_{X,Y}$ and $\theta_{X,Y}$ to find a region where the system is bistable, fixing hill coefficients as *m*=3 (see Middleton et al., 2009; Brown, 2012 for a more detailed justification). The parameter space was then further investigated by plotting bifurcation diagrams. Plotted in Fig. 4(a) are steady state solutions to (7) as functions of $\lambda_{\mathrm{AM},B}$. For an appropriate choice of parameters, the system is bistable, with stable steady states that can plausibly be interpreted as mesoderm and anterior mesendoderm. For small $\lambda_{\mathrm{AM},B}$ the system is monostable with the steady state representing the anterior mesendoderm. As $\lambda_{\mathrm{AM},B}$ increases, a fold bifurcation marks the onset of bistability and the appearance of the mesoderm stable steady state. For further increases of $\lambda_{\mathrm{AM},B}$, another fold bifurcation occurs and the system becomes monostable with the steady state representing mesoderm. We find that the concentrations of Mix and Brachyury at the anterior mesendoderm steady state are rather sensitive to changes in $\lambda_{\mathrm{AM},B}$, with Mix decreasing and Brachyury increasing as $\lambda_{\mathrm{AM},B}$ is increased. However, at the mesoderm steady state Mix and Brachyury concentrations are comparatively insensitive to changes in $\lambda_{\mathrm{AM},B}$ and levels of Goosecoid are insensitive at both of the stable steady states. In Fig. 4(b) and (c) bifurcation curves are plotted in the $\left(\lambda_{\mathrm{AM},B},\lambda_{A,M}\right)$ and $\left(\lambda_{\mathrm{AM},B},\lambda_{M,G}\right)$ parameter spaces, respectively. Bistability occurs for $\lambda_{\mathrm{AM},B}$, $\lambda_{A,M}$ and $\lambda_{M,G}$ sufficiently large.

### 4.2 Time-dependent solutions to the axolotl *in vitro* model

**Fig. 5:**
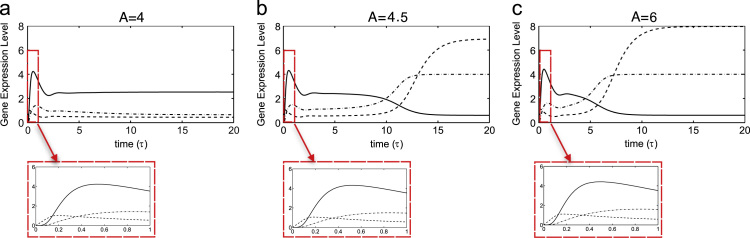
Numerical solutions of the axolotl *in vitro* model. The responses of Brachyury (thin solid line), Mix (dashed line) and Goosecoid (dot-dashed line) are shown. Parameters were chosen as in Table 2.

**Fig. 6:**
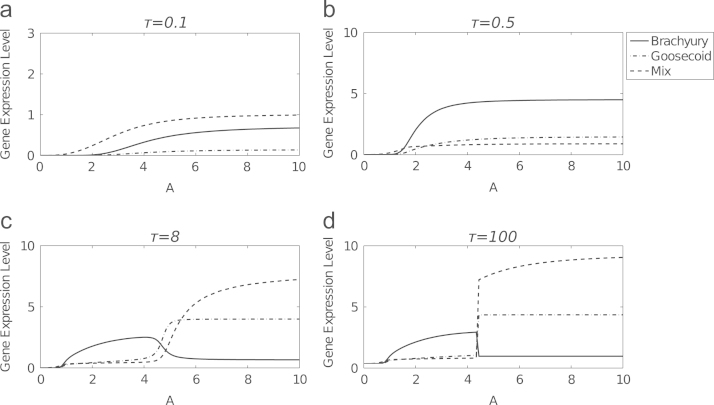
Numerical solutions of the axolotl *in vitro* model as functions of *A* for (a) *A*=4, (b) *A*=4.5, (c) *A*=6. The responses of Brachyury (thin solid line), Mix (dashed line) and Goosecoid (dot-dashed line) are shown. Parameters were chosen as in Table 2.

To compare solutions to the axolotl *in vitro* model with quantitative gene expression data from axolotl embryos and numerical results of the *Xenopus in vitro* model, we next explore time-dependent solutions to (7). The system evolves to the mesoderm or the anterior mesendoderm steady state dependent on the dose of Activin, as illustrated by numerical results in Fig. 5. A low dose of Activin causes the system to evolve to the mesoderm stable steady state (Fig. 5(a)). Mix expression precedes Brachyury expression before Brachyury becomes upregulated. At a high level of Activin (Fig. 5(c)) the system evolves to the anterior mesendoderm steady state, but initially we see similar behaviour to the time course given in Fig. 5(a). However, as time proceeds Mix and Goosecoid levels increase and Brachyury becomes downregulated. For an intermediate Activin dose (Fig. 5(b)) Brachyury is expressed for a longer time before levels of Mix and Goosecoid increase and Brachyury is repressed. These results are in qualitative agreement with experiments where Activin induces mesoderm and anterior mesendoderm in a dose dependent manner (see Section 2.2). Fig. 6 plots solutions to Eqs. (7) as functions of Activin dose (*A*). For *A* sufficiently small, the system stays at the trivial steady state. For *A* greater than some critical value, the system evolves to the mesoderm steady state and, as *A* is increased further, the system passes through another critical value and evolves to the anterior mesendoderm stable steady state.

In our time-course simulations, we find that at all doses of Activin there is a rapid increase in Brachyury expression occurring after Mix expression commences. Later the levels of Brachyury decrease and the system evolves to either the mesoderm or anterior mesendoderm steady state. Based on current experimental knowledge, this behaviour was unexpected. In whole embryos Mix expression precedes Brachyury, with Mix expressed from stage 8 and Brachyury expression commencing at stage 11 (Swiers et al., 2010). However, the only parameter sets we have found that yield bistability give this early peak of Brachyury expression.

The only difference between the axolotl model described above and the *Xenopus in vitro* model in Middleton et al. (2009) is the regulation of Brachyury by Mix. In *Xenopus* Mix negatively regulates Brachyury, but in axolotl Mix is required for the expression of Brachyury. Changing the sign of this interaction results in different parameter values driving bistability. Comparing the Activin dose response of the *Xenopus* model (Middleton et al., 2009) with the axolotl model (Fig. 6) shows qualitative differences in the expression profiles of the two models. In the *Xenopus* model, Mix and Goosecoid are not expressed at the mesoderm steady state, and Brachyury is not expressed at the endoderm steady state. In the axolotl model, although Brachyury is expressed at low levels compared with Mix or Goosecoid at the anterior mesendoderm steady state, it is expressed at a non-zero level. Similarly at the mesoderm steady state Mix and Goosecoid levels are nonzero, but at lower concentrations than Brachyury.

**Fig. 7:**
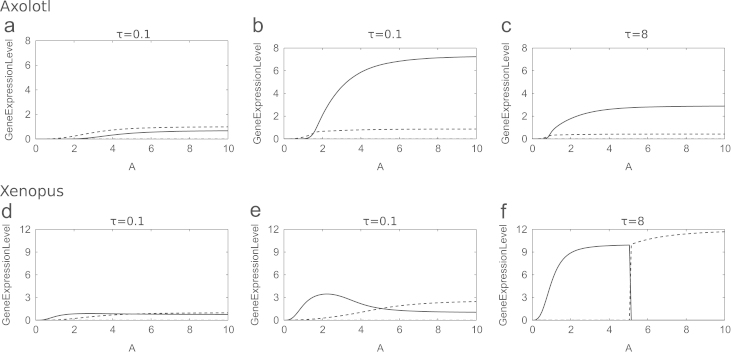
Numerical solutions of the *Xenopus in vitro* model (Middleton et al., 2009) and the axolotl *in vitro* model (7), in the absence of Goosecoid, as functions of *A*. The responses of Brachyury (thin solid line), Mix (dashed line) and Goosecoid (dot-dashed line) are shown. Parameters used are given in Table 2 for the axolotl model and as given in Middleton et al. (2009) for the *Xenopus*. Values of $\lambda_{M,G}$, $\theta_{G,G}$ and $\lambda_{\mathrm{XM},B}$ are higher in the axolotl model than in the *Xenopus* model, corresponding to higher rates of production of Mix and Brachyury and a higher threshold for Goosecoid negative autoregulation.

In the axolotl *in vitro* model, Goosecoid is an important factor as its negative regulation of Brachyury creating competition between Brachyury-expressing and Mix-expressing cells. In the *Xenopus in vitro* model, Goosecoid is not required for the formation of two opposing populations of cells. Fig. 7(a), (c), and (e) plots solutions to the *Xenopus in vitro* model in the absence of Goosecoid. Two distinct cell types form, corresponding to mesoderm and endoderm, with these fates being reached dependent on the dose of Activin. Corresponding solutions to the axolotl *in vitro* model in the absence of Goosecoid are plotted as functions of Activin concentration in Fig. 7(b), (d) and (f). For all concentrations of Activin, the system evolves to the mesoderm steady state, showing that Goosecoid is essential for the formation of a Mix up-regulated steady state.

## 5 The axolotl *in vivo* model

In this section we explore the behaviour of the axolotl *in vivo* model given by the system (9).

### 5.1 Steady state analysis

The steady state solutions of *β*-catenin and Nodal are determined by

(11) $$C^{⁎}=0,\;N^{⁎}=\psi H(\frac{N^{⁎}}{\theta_{N,N}}),$$

where $\psi =\lambda_{N,N}/\mu_{N}$. Eq. (11) is identical to the equation for the steady state of Nodal in the *Xenopus in vivo* model of Middleton et al. (2009). Eq. (11) is bistable with solutions *N*=0 and $N=N^{⁎}>0$ corresponding to downregulated and upregulated Nodal1, respectively. In the *N*=0 case steady states for *L*^⁎^, *E*^⁎^, *B*^⁎^, *M*^⁎^, *G*^⁎^ are given by

(12a) $$E^{⁎}=\frac{\lambda_{B,E}}{\mu_{E}}H(B^{⁎}),$$

(12b) $$B^{⁎}=\lambda_{E,B}H(E^{⁎}),$$

(12c) $$G^{⁎}=M^{⁎}=L^{⁎}=0.$$

Eqs. (12) form a bistable system, with steady states corresponding to FGF and Brachyury upregulating each other (i.e. mesoderm) and the trivial steady state (i.e. ectoderm). These steady states are identical to those found in the *Xenopus in vitro* model in the absence of Nodal. In the $N=N^{⁎}>0$ case, Nodal1 is maintained by positive autoregulation and steady states are defined by solutions to

(13a) $$L^{⁎}=\frac{\lambda_{N,L}}{\mu_{L}}H(N^{⁎}),$$

(13b) $$E^{⁎}=\frac{\lambda_{B,E}}{\mu_{E}}H(B^{⁎}),$$

(13c) $$B^{⁎}=\{\lambda_{E,B}H(E^{⁎})+\lambda_{\mathrm{NM},B}H(N^{⁎})H(M^{⁎})\}\{1-H(G^{⁎})\},$$

(13d) $$G^{⁎}=\frac{1}{\mu_{G}}\{\lambda_{M,G}H(\frac{M^{⁎}}{\theta_{M,G}})+\lambda_{L,G}H(\frac{L^{⁎}}{\theta_{L,G}})\}\{1-H(\frac{G^{⁎}}{\theta_{G,G}})\},$$

(13e) $$M^{⁎}=\frac{\lambda_{X,M}}{\mu_{M}}H(\frac{N^{⁎}}{\theta_{X,M}})\{1-H(\frac{B^{⁎}}{\theta_{B,M}})\}.$$

Solutions to (13) are equivalent to steady state solutions of the axolotl *in vitro* model (7) if we set $\lambda_{B,E}=0$ and $\lambda_{N,L}=0$ such that $E^{⁎}=L^{⁎}=0$. It therefore follows that (13) has stable steady states corresponding to mesoderm and anterior mesendoderm. The steady state equations (12) and (13) are independent of the concentration of *β*-catenin (*C*) since this decays to zero at the steady state. However *β*-catenin can determine cell fate in axolotl animal caps and we show that this is also the case in time-dependent solutions for the *in vivo* model in Section 5.2.

### 5.2 Time-dependent solutions

**Fig. 8:**
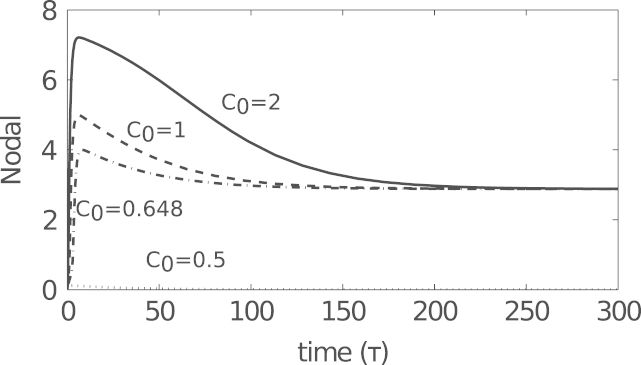
Numerical solutions to (9) subject to initial conditions (10) with *C*_0_ as shown above. For sufficiently large *C*_0_, *N* tends to *N*^⁎^, first overshooting this value. Parameters were chosen as in Table 3.

**Fig. 9:**
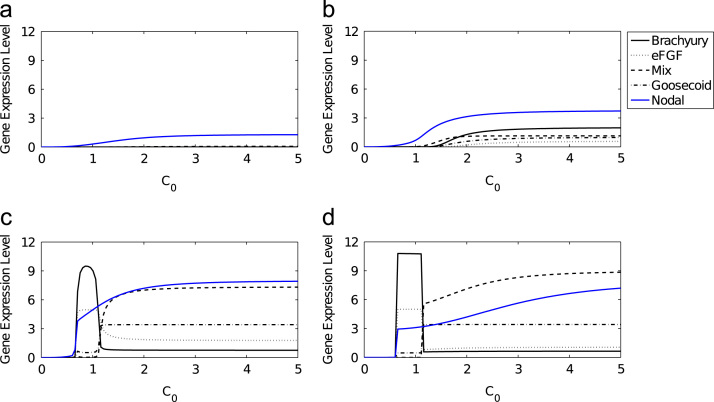
Numerical solutions of the axolotl model as functions of *C*_0_, for various values of *τ*. The response of Brachyury (thin solid line), eFGF (dotted line), Mix (dashed line), Goosecoid (dot-dashed line) and Nodal (blue solid line) are shown in response to an initial concentration of *β*-catenin. Parameters were chosen as in Table 3. (a) T=0.5, (b) T=1, (c) T=8 and (d) T=100. (For interpretation of the references to colour in this figure caption, the reader is referred to the web version of this paper.)

*β*-catenin is able to induce mesoderm and anterior mesendoderm in a dose dependent manner in axolotl animal caps, activating Nodal, which then acts on downstream targets (Chen, 2010). Numerical solutions to (9) show that for sufficiently large *C*_0_, *N* evolves to its non-trivial stable steady state, with solutions first overshooting this value. The extent of this overshoot is determined by *C*_0_, with larger overshoots for large *C*_0_ (Fig. 8). We now investigate time-dependent solutions to the full *in vivo* model, in particular the ability of *β*-catenin to induce mesoderm and anterior mesendoderm in a dose dependent manner. Solutions to (9) are plotted as functions of initial concentration of *β*-catenin (*C*_0_) in Fig. 9. These results suggest that, for an appropriate choice of parameters, *β*-catenin can determine the fate of a cell. For *C*_0_ small enough the system evolves to the mesoderm branch. As *C*_0_ is increased beyond some critical value, the system will evolve to the anterior mesendoderm branch. For *C*_0_ too small the system evolves to the trivial steady state where neither Mix nor Brachyury is expressed (i.e. ectoderm). These numerical results are in qualitative agreement with qPCR data collected from axolotl animal caps injected with *β*-catenin: at low doses of *β*-catenin animal caps become mesoderm (expressing Brachyury) and at higher doses cells become anterior mesendoderm (expressing Mix and Goosecoid) (Chen, 2010).

## 6 Discussion

In this paper we have developed and analysed single-cell models based on current knowledge of the axolotl mesendoderm GRN. The models describe the GRN downstream of Activin (*in vitro* model) and *β*-catenin (*in vivo* model), with both models having stable steady states corresponding to mesoderm (i.e. *Brachyury* expressing cells) and anterior mesendoderm (i.e. *Mix* and *Goosecoid* co-expressing cells). Here we give a summary of the key results presented in this paper and the outlook for future investigations.

A qualitative analysis of the *in vitro* and *in vivo* models found that both were able to reproduce experimental observations. The *in vitro* model reproduces the dose dependent induction of mesoderm and anterior mesendoderm, whereby low doses of Activin cause a cell to become mesoderm and high doses cause a cell to become anterior mesendoderm. Although the model evolves to steady states corresponding to mesoderm and anterior mesendoderm, it does not reproduce time courses of gene expression similar to those found in Activin injected animal caps. The *in vivo* model reproduces *β*-catenin dose response experiments (Chen, 2010) with a low concentration of *β*-catenin inducing mesoderm and a high concentration inducing anterior mesendoderm.

### 6.1 Regulatory circuits driving cell differentiation

**Fig. 10:**
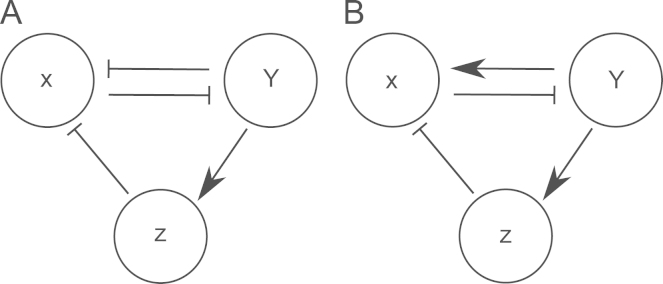
Gene regulatory networks which yield bistability. (A) Mutual negative regulation between X and Y drives differentiation of a cell to express either X or Y, with the indirect repression of X by Y (via Z) being dispensible. (B) An alternative network, where the indirect repression of X by Y (via Z) is necessary to drive differentiation of a cell to express Y.

A variety of regulatory network motifs have been shown to drive gene expression in biological systems. In particular, a mutual negative feedback loop between two transcription factors has been identified as a mechanism for the emergence of two distinct cell populations in response to a signal. A mutual negative feedback between *Mix* and *Brachyury* drives mesoderm and anterior mesendoderm formation in *Xenopus*, giving switch-like behaviour between the two cell fates. Here we explored an alternative mechanism for driving cell differentiation that consisted of the interactions of three transcription factors, namely *Mix*, *Brachyury* and *Goosecoid*. This model was shown to be bistable with steady states corresponding to mesoderm and anterior mesendoderm. A key difference between the *Xenopus* and axolotl network topologies was the requirement of *Goosecoid* for bistability. In the absence of *Goosecoid*, mesoderm and endoderm are steady states in the *Xenopus* model, while only mesoderm forms in the absence of *Goosecoid* in the axolotl model. Thus while Goosecoid is dispensible for forming Mix expressing cells in *Xenopus*, it appears to be required to form this population of cells in axolotl. This is a consequence of the change in the Mix/Brachyury relationship: in *Xenopus* both Mix and Goosecoid can repress the expression of *Brachyury*, while in axolotl Goosecoid is the only factor that can repress *Brachyury* in the mesendoderm GRN. Thus, while only two transcription factors are required in the mutual negative regulation network to form a bistable switch, three transcription factors are required in our alternative model, based on the axolotl mGRN (Fig. 10).

The maternal factors required to induce mesoderm and endoderm also vary between *Xenopus* and axolotl. In *Xenopus* VegT is present in a gradient running from the vegetal to animal pole, while *β*-catenin is expressed dorsally and both of these factors have been shown to be important in mesendoderm formation. In *Xenopus* investigations of mathematical models of the mesendoderm GRN show that mesoderm and anterior mesendoderm form in regions determined by the concentrations of VegT and *β*-catenin (Middleton et al., 2009). In axolotl, VegT does not function in mesendoderm formation (Chen, 2010) and *β*-catenin is expressed dorsally. We show in our mathematical model how mesoderm and anterior mesendoderm can form dependent on the dose of *β*-catenin. This is a surprising result since in *Xenopus* VegT rather than *β*-catenin provides the main initial positional information to drive mesendoderm formation.

To summarise, whilst studying the differences in mesendoderm formation in *Xenopus* and axolotl, we found that several mechanisms thought to be central in driving mesendoderm formation in *Xenopus* are not present in axolotl (e.g. mutual inhibition of Mix/Brachyury and the graded distribution of VegT). By studying the axolotl mesendoderm GRN using mathematical models we have improved our understanding of the mechanisms via which mesendoderm is formed in axolotl. Despite these differences in the mechanisms by which mesendoderm forms, the primary germ layers form in the correct regions in both *Xenopus* and axolotl.

### 6.2 Why have different network topologies for mesoderm and anterior mesendoderm evolved?

The mesendoderm GRN was first studied in the axolotl in anticipation of a simpler structure for the mesendoderm GRN than found in *Xenopus*, because of the presence of fewer *Mix* and *Nodal* genes. However, fundamental differences have emerged in the topology of the GRN in axolotl compared with *Xenopus*, which were initially surprising since it was reasonable to assume that the mechanisms underlying mesendoderm formation in the two amphibians would be similar (Swiers et al., 2010). Comparison with data from mouse suggests that it is the axolotl topology, not *Xenopus*, which best reflects mammalian mesoderm specification (Swiers et al., 2010). The differences in the mechanisms of mesendoderm formation in axolotl and *Xenopus* become less surprising when considering other aspects of embryo development. For example, the mechanisms underlying the regulation of pluripotency^1^, the method by which primordial germ cells (PGCs) arise and the mechanisms of gastrulation are the same in axolotl and mammals, but different in *Xenopus* (Johnson et al., 2011, 2003; Kaneda and Doi Motoki, 2012). Most likely, the differences in the early development of frogs and axolotls arise as a consequence of constraint release due to a change in the location of PGC formation. In axolotl, PGCs are induced in the mesoderm where changes in the mesendoderm GRN would eliminate PGCs; but in *Xenopus* PGCs are predetermined in the endoderm such that changes in the mesendoderm GRN do not eliminate PGCs, thus mechanisms of mesendoderm formation are able to evolve in *Xenopus* without altering the specification of the PGCs (Johnson et al., 2011).

### 6.3 Future outlook

The qualitative analysis of the axolotl mesendoderm GRN downstream of Activin provided several useful insights into the behaviour of the network. However, to develop a model with biologically valid parameter values, more experimental data are required. These data could be in the form of more detailed time courses or a detailed Activin dose response curve. Data on the behaviour of the network in response to perturbations would also aid the development of a model which is able to reproduce experimental observations fully. Once the axolotl mesendoderm GRN has been fully explored and quantified downstream of Activin, this knowledge would aid the study of the network in response to *β*-catenin. By collecting experimental data downstream of *β*-catenin, parameter values of the axolotl *in vivo* model can be estimated. Furthermore experimental data on the distribution of maternal transcripts of *β*-catenin in whole embryos, and how this overlaps with Mix, Brachyury and Goosecoid, could be used in multicellular mesendoderm models. Another future direction for developing multicellular models is to incorporate cell growth and the movements of gastrulation. Finally, Nanog, a key regulator of pluripotency, has been identified in the axolotl, but no ortholog exists in the genome of *Xenopus* (Dixon et al., 2010; Hellsten et al., 2010). There is evidence that Nanog may play a role in the specification of mesoderm in mammals. Thus, *Xenopus* must have evolved mechanisms to compensate for the loss of Nanog in its GRN. Exploring the role that Nanog plays in the establishment of the mesoderm and endoderm and how it integrates with the axolotl mGRN will be of great future interest.

To conclude, this work has given insight into the mechanisms by which mesendoderm forms, identifying the axolotl as a suitable model organism for studying a simplified mesendoderm GRN, and comparing the mechanisms of mesendoderm formation in axolotl with those in *Xenopus*. Furthermore, the directions for future studies, just noted, both experimental and theoretical, would aid understanding of mammalian development.

## Figures and Tables

**Table 1:** A Summary of genes present in the axolotl mGRN, their type (signal or transcription factor) and notation used in the mathematical model.

Protein	Signal or TF	Protein concentration
*β*-catenin	Signal	*C*
Nodal1	Signal	*N*
Activin	Signal	*A*
Mix	TF	*M*
Brachyury	TF	*B*
Goosecoid	TF	*G*
eFGF	Signal	*E*
Lim1	TF	*L*

**Table 2:** Dimensionless parameter values used to solve the axolotl *in vitro* model given in (7). Parameters were selected such that (7) is bistable with steady states corresponding to mesoderm and anterior mesendoderm, and so that the system evolves to these steady states dependent on the concentration of Activin (A>0).

Variable	Parameter	Value	Variable	Parameter	Value
*M*	$\lambda_{A,M}$	11	*B*	$\lambda_{\mathrm{AM},B}$	40
	$\theta_{A,M}$	3	*G*	$\lambda_{M,G}$	8
	$\theta_{B,M}$	1		$\theta_{M,G}$	1
	All *μ*	1		$\theta_{G,G}$	4
	all *m*	3			

**Table 3:** Dimensionless parameter values used to obtain numerical results in the axolotl *in vivo* model. Parameters were selected such that (9) is bistable with steady states corresponding to mesoderm and anterior mesendoderm, and so that the system evolves to these steady states dependent on the concentration of *β*-catenin ($C_{0}>0$).

Variable	Parameter	Value	Variable	Parameter	Value
*C*	$\mu_{C}$	0.01	*M*	$\lambda_{X,M}$	12

*N*	$\lambda_{N,N}$	3		$\theta_{X,M}$	3
	$\theta_{N,N}$	1		$\theta_{B,M}$	1
	$\lambda_{C,N}$	1	*G*	$\lambda_{M,G}$	8
	$\lambda_{C,N2}$	1		$\theta_{M,G}$	1

*B*	$\lambda_{X,B}$	6		$\lambda_{L,G}$	1
	$\lambda_{E,B}$	12		$\theta_{G,G}$	3

*E*	$\lambda_{B,E}$	12	*L*	$\lambda_{N,L}$	1
	All other *μ*	1		$\theta_{N,L}$	1
	All *m*	3			

## Appendix A Materials and methods

Animal cap experiments were carried out as described in the supporting information.

Application 1

## Footnotes

1 Pluripotency refers to the ability of a cell to give rise to any cell type of the adult organism.
