# Supplementary material for: Two different network topologies yield bistability in models of mesoderm and anterior mesendoderm specification in amphibians
Source: J Theor Biol. 2014 Jul 21;353(100):67–77. doi: 10.1016/j.jtbi.2014.03.015 (PMC4029075; doi:10.1016/j.jtbi.2014.03.015)
Supplement: Application 1 [file mmc1.pdf]

Supporting Material - Experimental Materials and Methods

L. E. Brown[a,1,*], J. R. King[b], M. Loose[c]

[a] MyCIB, School of Biosciences, University of Nottingham, Sutton Bonington, LE12 5RD, UK
[b] School of Mathematical Sciences, University of Nottingham, University Park, Nottingham, NG7 2RD,UK
[c] Centre for Genetics and Genomics, University of Nottingham, Queen's Medical Centre, Nottingham, NG7 2UH, UK

1. Axolotls

Natural matings were established as described in [1]. One or two cell embryos were placed in 1 MBS + 4 % Ficoll (Sigma) and antibiotics, then injected in the animal hemisphere with 2 4 nl injections (one per blastomere in two cell embryos). Embryos were staged according to [2], which are approximately equivalent to the stages of *Xenopus* in [4]. Animal caps were cut from embryos at stage 9 and cultured for 48 hours.

2. Quantitative RT - PCR

qPCR was performed using the ABI 7500 Sequence Detection System (Applied Biosystems) with TaqMan probes and primers as detailed below. RNA was isolated from a minimum of 10 animal caps. qRT-PCR data were analysed by the comparative CT method [3]. Validation experiments were carried out on a 4-fold dilution series of cDNAs from 1 to 1/256 to ensure the PCR efficiencies of the target and endogenous reference, (ODC), were approximately equal. The

* Corresponding Author
Email addresses: laura.brown@slcu.cam.ac.uk (L. E. Brown), john.king@nottingham.ac.uk (J. R. King), matt.loose@nottingham.ac.uk (M. Loose)
[1] Current Address: Sainsbury Laboratory Cambridge University, Cambridge, CB2 1LR, UK

Preprint submitted to J Theor. Biol. *March 6, 2014*

data were analysed in excel (Microsoft) and graphs were plotted of the ratio of gene expression relative to uninjected animal caps. Error bars are one standard deviation of the sample.

2.1. Primers and probes

Primers and probes were designed using Primer Express version 3.0 software (Applied Biosystems) according to manufacturer's instructions. Probes (Sigma) are dual-labelled fluorogenic probes (5' FAM; 3' TAMRA) and HPLC purified.

	Forward (5'-3')	Reverse (5'-3')
AxNodal1	CCCAGTGGATGAAACGTTCAG	GGGTCGGGTGGTACAGCTT
AxBrachyury	CATTGACCACATGTACCAATTGC	GATCAAGGGTCAATCGTGAGTTC
AxMix	GTCCAGGATCCAGGTCTGGTT	GCTTCTGGGTGGATTTGATTTATAA
AxFGF8	TGCAGGTCCTTGGCAACAA	AAGGTGTCCGTTTCCACAATTAA
AxSox17	TGGATACGACGCTCCACAGA	CTCCCTGTAGTGGCCGATGT
AxGsc	GCCTCTTCCAGGAGACCAAGT	TGGCTCTGCGGTTCTTGAAC
AxODC	ATGCCCGTCATGAGTAGTACCA	CCCGGACCCAGGTTACG
AxNcam	TGAATGTCGTTCAACGTGAGAGA	AAGAAAAGACTCTGGATGGACGTATC
	Probe (5'-3')	
AxNodal1	CGACGAATCATGCCTACATGCAGAGC	
AxBrachyury	TACCCATAGTTCTTTTGTGCAGCATCCACG	
AxMix	AATAGGCGTGCCAAGTCCCGCC	
AxFGF8	ACGGCGACTCGCACGCCA	
AxSox17	CATGAGCAGCAGTTCCAGCAGGACAAC	
AxGsc	CACCCGAGAGCAGCTGGCCC	
AxODC	GACAGTTCCAAGGTTTCATTCAATTGCTG	
AxNcam	AGACCCTGGCGTGACTGCTCACCA	

2.2. Microscopy and photography

Animal caps were visualised under Nikon SMZ 1500 microscopes. Photographs were taken using a Nikon DXM 1200F camera.

References

[1] J.B. Armstrong and G.M. Malacinski. *Developmental biology of the axolotl.* Oxford University Press, 1989.

[2] N.P. Bordzilovskaya and T.A. Dettlaff. Table of stages of the normal development of axolotl embryos and the prognostication of timing of successive developmental stages at various temperatures. *Axolotl Newsl.*, 7:2–22, 1979.

[3] K.J. Livak and T. D. Schmittgen. Analysis of relative gene expression data using real-time quantitative pcr and the 2(-δ δ c(t)) method. *Methods*, 25(4):402 – 408, 2001.

[4] P.D. Nieuwkoop and J. Faber. *Normal table of Xenopus laevis (Daudin).* Garland Publishing Inc, New York, 1994.
